# *Salmonella* Typhimurium PagP- and UgtL-dependent resistance to antimicrobial peptides contributes to the gut colonization

**DOI:** 10.1371/journal.pone.0190095

**Published:** 2017-12-21

**Authors:** Ryosuke Goto, Tsuyoshi Miki, Nao Nakamura, Mayuka Fujimoto, Nobuhiko Okada

**Affiliations:** Department of Microbiology, School of Pharmacy, Kitasato University, Tokyo, Japan; Institut National de la Recherche Agronomique, FRANCE

## Abstract

Mucosal barrier formed by cationic antimicrobial peptides (CAMPs) is believed to be crucial for host protection from pathogenic gut infection. However, some pathogens can develop resistance to the CAMPs to survive in hosts. *Salmonella enterica* is a common cause of acute diarrhea. During the course of this disease, the pathogen must continuously colonize the gut lumen, which contains CAMPs. However, it is incompletely understood whether the resistance of *Salmonella* strains to CAMPs contributes to the development of gut infections. PhoPQ two-component system-dependent lipid A modifications confer resistance to CAMPs in *S*. *enterica* serovar Typhimurium. Therefore, we introduced mutations into the PhoPQ-regulated genes in an *S*. Typhimurium strain, obtaining *pagP ugtL* and *pmrA* mutant strains. Each mutant strain demonstrated a distinct spectrum of the resistance to CAMPs. Using streptomycin mouse model for *Salmonella* diarrhea, we show that the *pagP ugtL*, but not *pmrA*, mutant strain had a gut colonization defect. Furthermore, the *pagP ugtL*, but not *pmrA*, mutant strain had decreased outer membrane integrity and susceptibility to magainin 2, an alpha-helical CAMP. Taken together, the PagP- and UgtL-dependent resistance to CAMPs was demonstrated to contribute to sustained colonization in the gut. This may be due to the robust outer membrane of *S*. Typhimurium, inducing the resistance to alpha-helical CAMPs such as α-defensins. Our findings indicate that the development of resistance to CAMPs is required for the *S*. Typhimurium gut infection.

## Introduction

Resistance to cationic antimicrobial peptides (CAMPs) represents a major virulence factor in the infectious stages of *Salmonella enterica* serovar Typhimurium (*S*. Tm). The PhoPQ-dependent modifications of the outer membrane lead to the development of *S*. Tm resistance [1, 2]. Lipid A modifications induce reduction of the anionic charge on the bacterial surface and a decrease in the bacterial surface membrane fluidity, allowing its escape from CAMP binding and membrane insertion. Therefore, *S*. Tm can survive and colonize the gut even in the presence of CAMPs.

The PhoPQ is a two-component system that controls the transcript levels of many genes associated with the resistance to CAMPs. The sensor PhoQ can be activated by several inducing factors, such as low Mg^2+^ levels [3], mildly acidic pH [4, 5], or CAMPs [6], which results in the phosphorylation of its cognate response regulator PhoP. The phosphorylated PhoP represents the active form of this molecule, able to bind to promoter regions, stimulating or repressing gene transcription. For example, the PhoP activation increases the expression of *pag* genes, such as *pagP* and *ugtL*, conferring resistance to defensin [7].

A number of studies on the PhoPQ-mediated resistance of *S*. Tm strains to antimicrobial peptides including CAMPs focused on the survival within macrophages and during systemic infections [8–16]. Despite the fact that CAMPs are expressed and secreted into the gut lumen, and subsequently specifically stimulate the PhoQ sensor [6], little is known whether the resistance to CAMPs is involved in the gut infection, *i*.*e*., pathogen colonization in the gut. Therefore, we analyzed the role of PhoPQ-mediated resistance to CAMPs during *Salmonella*-induced diarrhea.

In this study, we focused on two distinct types of resistance to CAMPs based on different PhoPQ-dependent lipid A modifications by PagP and UgtL, or PmrA. PagP modifies lipid A molecules by adding palmitate, which results in the formation of lipid A with seven acyl chains and one palmitoyl group [17]. UgtL is a PhoQ accessory protein [18], which contributes to the formation of monophosphorylated lipid A [19]. Although each PagP- and UgtL-dependent lipid A modification contributes to the resistance of *S*. Tm to CAMPs, the synergy of these modifications confers more potent resistance than each individual modification [19]. PhoP can induce the production of lipid A with alternative modifications through activation of the PmrA response regulator [7]. PmrA-dependent lipid A modifications, such as the addition of 4-amino-4-deoxy-L-arabinose (Ara4N) [20, 21] and phosphoethanolamine (pEtN) [21] to the phosphate groups of lipid A, can reduce the negative charge of the bacterial cell surface, affecting the electrostatic interactions with CAMPs. Therefore, *pmrA* mutants are more susceptible to polymyxin B [7].

Here, we studied the role of the resistance to CAMPs during *S*. Tm colonization in the gut using the streptomycin mouse model of the acute *Salmonella* diarrhea [22, 23]. Our results suggest that the development of resistance to CAMPs is necessary for the sustained *S*. Tm colonization in the gut. The findings described here promote a better understanding of the role of resistance to CAMPs in pathogen colonization in the gut.

## Results and discussion

### PagP- and UgtL-, or PmrA-dependent resistance to antimicrobial peptides

To understand the resistance to CAMPs in *Salmonella*-induced diarrhea, we focused on two types of lipid A modifications induced by PagP and UgtL or PmrA. Previously, different lipid A modifications were shown to confer different types of *S*. Tm resistance to CAMPs [7, 19]. Here, we determined the minimum inhibitory concentrations (MICs) of two CAMPs, polymyxin B and protamine, towards *pagP ugtL* mutant (T230) or *pmrA* mutant (T141) (Table 1). Mutations in the *pagP* and *ugtL* genes were shown to attenuate the ability of *S*. Tm cells to resist protamine, but not polymyxin B. In contrast, *pmrA* mutant cells showed a decrease in the resistance to polymyxin B, but not to protamine. Furthermore, we performed an *in vitro* killing assay. Although T230 seemed to be killed by polymyxin B to some extent, similar results were obtained in this experiment (S1 Fig). These results confirmed that different lipid A modifications allow the resistance of cells to specific types of CAMPs. Therefore, we used these strains in further experiments in this study.

**Table 1:** Minimal inhibitory concentrations (MIC) of cationic antimicrobial.

Strain	MIC (μg/ml)	
	Polymyxin B	Protamine
*S*. Tm SL1344 (wild type)	3.67	64.0
T230 (*pagP ugtL*)	3.00	44.0
T141 (*pmrA*)	2.20	61.3

MIC is described as the average (μg/ml) of at least six experiments.

### *S*. Tm cells harboring mutations in the *pagP* and *ugtL* or *pmrA* genes can colonize the gut and induce the inflammatory responses

**Fig 1:**
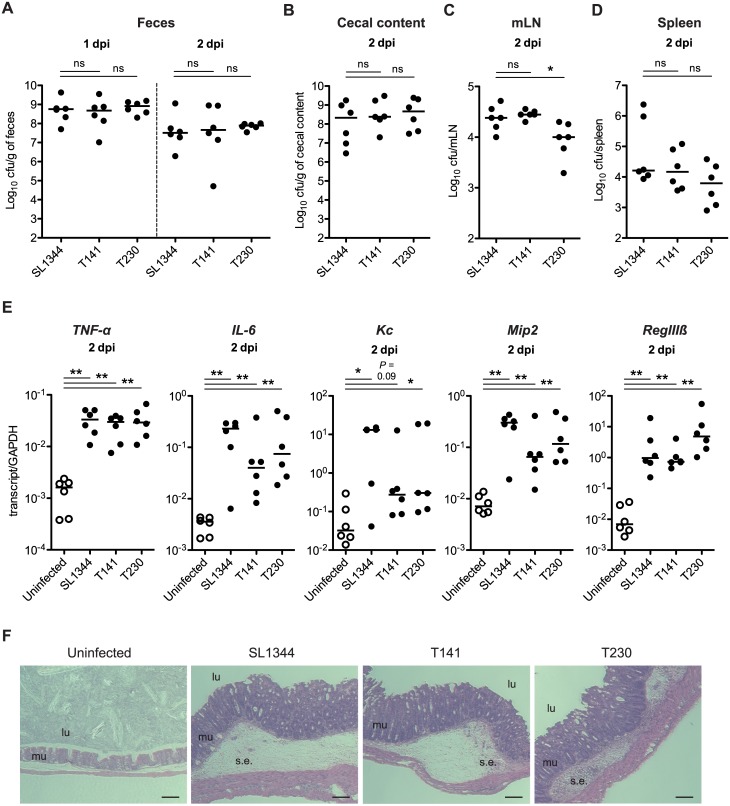
*S*. Tm *pmrA* mutant and *pagP ugtL* mutant are capable of triggering gut colonization and inflammation. Three groups of streptomycin-pretreated C57BL/6 mice (n = 6 per group) were infected for 2 days with the wild-type *S*. Tm strain (SL1344), Δ*pmrA*::*cat* strain (T141) or Δ*pagP ugtL*::*kan* strain (T230) by gavage. At 1 and 2 days post infection, *S*. Tm loads in the feces were determined. At 2 days postinfection (dpi), mice were sacrificed and *S*. Tm colonization of cecum, mLN, and spleen were examined. (A to D) The number of *S*. Tm CFU in different organs (feces (A), cecal content (B), mLN (C), spleen (D)). Horizontal bars, median; ns, not significant (P ≥ 0.05); *P < 0.05; Mann-Whitney U test. (E) Transcript levels of inflammatory cytokines (*TNF-α* and *IL-6*), chemokines (*Kc* and *Mip2*) and antimicrobial effector (*RegIIIβ*) in cecal tissue. Horizontal bars, median; *P < 0.05; **P < 0.01; ***P < 0.001; Mann-Whitney U test. (F) Hematoxylin/eosin (H&E) stained cecal section (100×). Scale bar, 20 μm. lu, lumen; mu, mucosa; s.e., submucosal edema.

To demonstrate the role of lipid A modification-mediated resistance to CAMPs in the acute diarrhea model induced by *S*. Tm, we performed gut infection experiments using the streptomycin mouse model [22] and the CAMP-susceptible *S*. Tm strains. Streptomycin-pretreated C57BL/6 mice were infected with the *S*. Tm wild-type strain (SL1344), the *pagP ugtL* mutant cells (T230), or the *pmrA* mutant cells (T141) via the oral route (5 × 10^7^ CFU in total, by gavage). At day 1–2 post infection (p.i.), we determined the bacterial loads in feces. Furthermore, at day 2 p.i., the mice were sacrificed and the colonization of the cecal lumen, the mesenteric lymph node (mLN), and spleen was analyzed. The analysis of feces showed that all analyzed strains had similar colonization levels at day 1 and 2 p.i. (Fig 1A). Colonization levels in the cecal content and the spleen did not significantly differ between the strains (Fig 1B and 1D). In mLN, the mutant strain T230 colonization levels were shown to be significantly reduced (Fig 1C). Furthermore, the expression of inflammatory cytokines (*TNF-α* and *IL-6*), chemokines (*Kc* and *Mip2*), and antimicrobial effector (*RegIIIβ*) was shown to be induced in mice infected with the *S*. Tm strains, compared with that in the controls (Fig 1E). Histopathological analyses of the cecum tissue demonstrated that the mice infected with the analyzed strains featured significant levels of gut inflammation (Fig 1F). These results indicate that PagP- and UgtL-, or PmrA-dependent lipid A modifications are not required for *S*. Tm growth at the initial infectious stage and the induction of inflammation during the development of acute colitis. Moreover, the resistance to CAMPs was demonstrated to play no role in the induction of *Salmonella*-associated diarrhea.

### PagP and UgtL, but not PmrA, contribute to sustained *S*. Tm colonization in the gut

**Fig 2:**
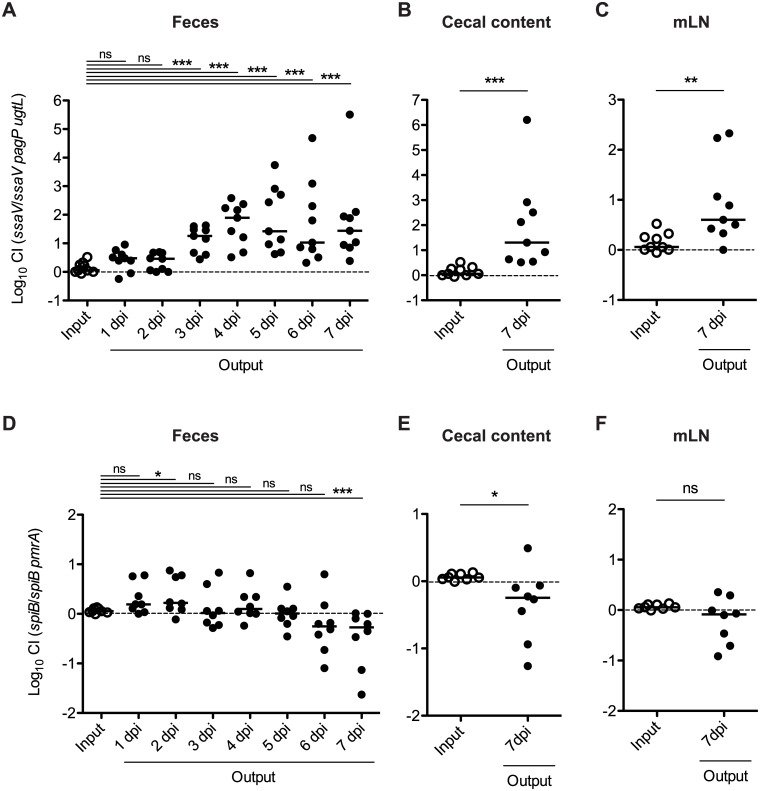
*S*. Tm *pagP ugtL* mutant, but not *pmrA* mutant, is impaired in the sustained gut colonization in competitive infection. Streptomycin-pretreated C57BL/6 mice (n = 8 or 9) were infected for 7 days with a 1:1 mixture (total 1 × 10^8^ CFU intragastrically) of *ssaV* mutant and *ssaV pagP ugtL* mutant, or *spiB* mutant and *spiB pmrA* mutant. *S*. Tm loads in feces (A and D), cecal content (B and E), and mLN (C and F) were determined by selective plating. Competitive infection indices (CI) were determined over 7 days. Horizontal bars, median; ns, not significant (P ≥ 0.05); *P < 0.05; **P < 0.01; ***P < 0.001; Mann-Whitney U test.

Next, we investigated whether the resistance to CAMPs contributes to sustained *S*. Tm colonization in the gut. Attenuated *S*. Tm mutant cells, harboring type III secretion system 2 (ttss-2) mutations, allow investigation of the later stages of gut infections [24, 25]. Streptomycin-treated mice were infected with a 1:1 bacterial mixture of ttss-2 mutant T145 (Δ*ssaV*::*cat*) and T216 (Δ*ssaV* Δ*pagP* Δ*ugtL*::*kan*), or ttss-2 mutant T118 (Δ*spiB*::*kan*) and T223 (Δ*spiB* Δ*pmrA*::*cat*) via the oral route. Bacterial loads in murine feces were monitored daily for the next 7 days. T216 cells showed a colonization defect by day 3 p.i. (competitive index, C.I. 14.4; Fig 2A). Subsequently, until the finalization of the experiment at day 7 p.i., the defective colonization was observed. In contrast, T223 did not show any colonization defects, and this strain was shown to colonize the gut more efficiently than the parental strain T118 (C.I. at day 7 p.i. 0.45; Fig 2D). Similar results were obtained by analyzing the cecal content and mLN (Fig 2B, 2C, 2E and 2F).

To reveal whether deletions of both *pagP* and *ugtL* genes are required for the colonization defect of T216 strain, we further performed mouse infection experiments using mixed *S*. Tm strains: T145 (Δ*ssaV*::*cat*) and T227 (Δ*ssaV* Δ*pagP*::*kan*), or T145 and T228 (Δ*ssaV* Δ*ugtL*::*kan*). In contrast to T216, T227 and T228 cells displayed no colonization defect at day 1, 3, 5, and 7 p.i. (S2 Fig). Rather, T228 cells colonized in the gut more efficiently than the parental strain T145 at day 1, 3, and 5 p.i. (S2 Fig). Furthermore, a colonization defect of T227 or T228 cells in the cecal content and mLN was not found (S2 Fig).

Collectively, these results suggest that the PagP- and UgtL-, but not PmrA-, resistance to CAMPs contributes to the sustained colonization of the gut by *S*. Tm.

### PagP and UgtL, but not PmrA, contribute to the construction of a robust outer membrane barrier

**Fig 3:**
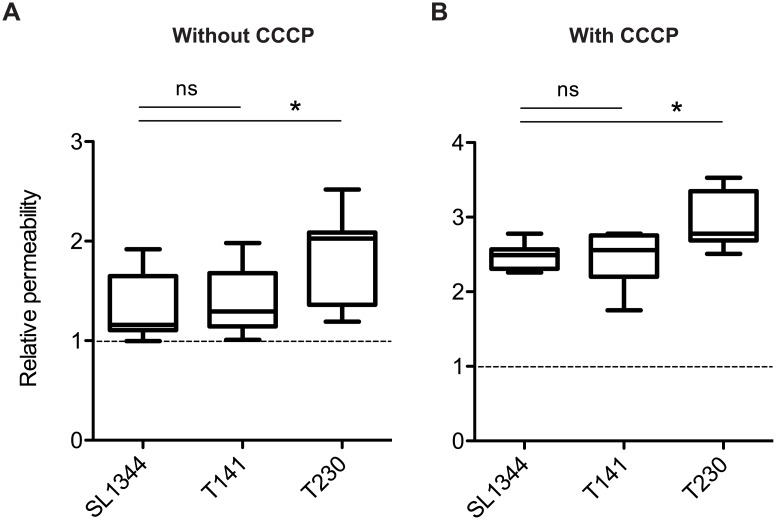
*S*. Tm *pagP ugtL* mutant, but not *pmrA* mutant, is impaired in the outer membrane integrity. The outer membrane permeability of wild-type *S*. Tm strain (SL1344), Δ*pmrA*::*cat* strain (T141) or Δ*pagP ugtL*::*kan* strain (T230) were measured using the EtBr influx assay [26, 27]. Outer membrane permeability was evaluated by comparing the sample without *S*. Tm, meaning that more than 1 indicates outer membrane permeability was increased. Two reactive conditions were performed without CCCP (A) or with CCCP (B). Box & whiskers plot: the whiskers denote minimum and maximum, and the black bars indicate medians. ns, not significant (P ≥ 0.05); *P < 0.05; Mann-Whitney U test.

PhoPQ-dependent lipid A modifications provide the robust outer membrane barrier of *S*. Tm [1, 26]. Therefore, we next examined whether the PagP- and UgtL-dependent lipid A modifications are required for the generation of the robust outer membrane. To assess outer membrane barrier integrity, we used the ethidium bromide (EtBr) influx assay [26, 27]. EtBr is a well-known DNA-intercalating fluorescent dye that cannot pass through the outer membrane of Gram-negative bacterium. However, if the outer membrane integrity is compromised, EtBr can pass through the outer membrane, and subsequently traverse the cytoplasmic membrane and reach the cytosol [26]. In the bacterial cytosol, EtBr rapidly binds to intracellular nucleic acids, leading to an increase in fluorescence signal. We did not observe an increase in the fluorescence intensity in the *pmrA* mutant cells (T141), in comparison with that in the wild-type *S*. Tm strain cells (SL1344) (Fig 3A). In contrast, the enhanced signal intensity was observed in the *S*. Tm T230 cells harboring *pagP* and *ugtL* mutations (Fig 3A). Furthermore, carbonyl cyanide *meta*-chlorophenylhydrazone (CCCP) was added to the bacterial mixture to inhibit the bacterial efflux pumps, and this allowed us to detect enhanced fluorescence signals [26]. Here, the obtained results were similar to those previously described (Fig 3B). Collectively, these results suggest that the PagP- and UgtL-dependent lipid A modifications, but not PmrA-dependent modifications, contribute to the robustness of the bacterial outer membrane.

### PagP and UgtL, but not PmrA, contribute to the resistance to magainin 2

**Fig 4:**
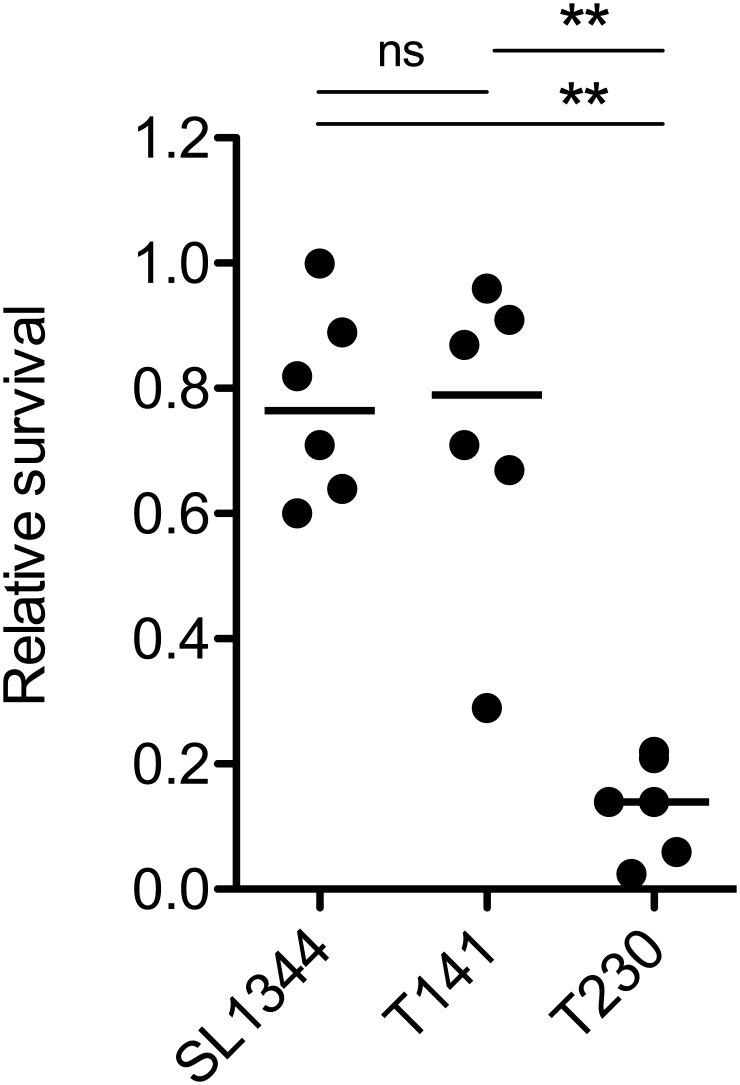
*S*. Tm *pagP ugtL* mutant, but not *pmrA* mutant, is impaired in the resistance to magainin 2. Wild-type *S*. Tm strain (SL1344), Δ*pmrA*::*cat* strain (T141) or Δ*pagP ugtL*::*kan* strain (T230) from the mid-logarithmic growth phase was incubated with magainin 2 (128 μg/mL) for 30 min at 37°C. Bacterial survival was quantified by dilution-plating. Bacterial killing effect was evaluated by comparing the sample without magainin 2, meaning that less than 1 indicates killing effect was increased. Horizontal bars, median; ns, not significant (P ≥ 0.05); *P < 0.05; **P < 0.01; ***P < 0.001; Mann-Whitney U test.

In the gut, alpha-helical CAMPs (α-CAMPs) called human defensin 5 (HD-5) or cryptdin represent the predominant CAMPs in humans or mice [28]. Therefore, we hypothesized that the colonization defect of *pagP ugtL* mutant in the gut is attributable to the α-CAMP-mediated removal of *S*. Tm cells. We thus investigated whether the PagP- and UgtL-dependent lipid A modifications contribute to the development of resistance to magainin 2, an α-CAMP. The wild-type *S*. Tm strain (SL1344) and the *pmrA* mutant strain (T141) were shown to be resistant to the magainin 2-mediated killing (Fig 4). In contrast, the *pagP ugtL* mutant strain (T230) was more sensitive to the α-CAMP effects, in comparison with SL1344 and T141 strains (Fig 4). We next performed the complementation experiment to compensate for the loss of both *pagP* and *ugtL* genes. T145 (Δ*ssaV*::*cat*) or T343 (Δ*ssaV* Δ*pagP* Δ*ugtL*) or T364 (Δ*ssaV* Δ*pagP* Δ*ugtL* harboring pMW118 and pACYC184), or T365 (Δ*ssaV* Δ*pagP* Δ*ugtL* harboring pMW118 encoding *pagP* and pACYC184 encoding *ugtL*) were subjected to an *in vitro* killing assay with magainin 2. T343 and T364 strains were susceptible to magainin 2 in comparison with T145 (S3 Fig). In contrast, T365 strain was not killed by magainin 2 (S3 Fig), indicating that complementation with the plasmids encoding *pagP* or *ugtL* respectively restored the impaired resistance against magainin 2. The obtained results suggest that the attenuated resistance to α-CAMPs may be attributable to the reduced levels of Δ*pagP* Δ*ugtL*::*kan* cells (T230) in the gut.

## Conclusions

The gut *Salmonella* lipid A may be modified by the PhoPQ two-component system, since the CAMPs can directly stimulate the PhoQ sensor, leading to the PhoP-dependent gene expression [6, 29]. In the intestinal lumen, the PhoPQ-regulated genes (*pagP*, *pagJ*, and *pmrH*) were shown to be activated in a PhoPQ-dependent manner [30, 31]. Therefore, the PhoPQ-dependent lipid A modifications in the gut most likely are responsible for the resistance to CAMPs, allowing *Salmonella* cells to survive. Indeed, in the study by Woo *et al*., *S*. Tm *phoP* mutant cells appeared to be unable to persist the murine gut, indicating that the PhoPQ system is involved in the *S*. Tm colonization in the gut [32]. Furthermore, a number of previous reports showing that the CAMPs play the protective role during oral *Salmonella* infections [11, 14, 16, 20, 33]. However, in most of these studies, antimicrobial effects of CAMPs were evaluated by investigating lethal systemic infections. Furthermore, it is unlikely that diarrhea was induced in the infected mice since the streptomycin mouse model was not employed in these studies. Therefore, the resistance of *S*. Tm to CAMPs during gut infections, and especially during the activation of host inflammatory response, was unclear. Here, we used the streptomycin mouse model to induce gut inflammation in order to elucidate the role of bacterial resistance to CAMPs during *Salmonella*-induced diarrhea. The findings presented here indicate that certain lipid A modifications by PagP and UgtL may contribute to sustained colonization in the gut. Furthermore, it may be concluded that these effects correlate with the presence of a robust outer membrane with low fluidity, which prevents the insertion of α-CAMPs.

More recently, UgtL has been shown to be a PhoQ accessory protein activating the PhoPQ-regulated genes in response to certain conditions [18]. In acidic pH, lack of the *ugtL* gene results in reduced expression levels of the PhoPQ-regulated genes. Thus, UgtL-dependent PhoQ activation appears to be restricted to the specific condition, i.e., acidic pH, but not the presence of antimicrobial peptides [18]. Further studies will be needed to clarify precise mechanisms of the additive and synergy effects with PagP and UgtL on the *S*. Tm gut colonization.

In conclusion, the obtained data demonstrate for the first time that the robust outer membrane conferring the resistance to α-CAMPs contributes to the *S*. Tm colonization in the gut. We demonstrated that the development of resistance to α-CAMP is required for *S*. Tm colonization in the gut. Future studies, using CAMP knockout mice, may be necessary to establish the causal link between the resistance to CAMPs and the *S*. Tm colonization in the gut.

## Materials and methods

### Bacterial strains and plasmids

All strains used in this study are the derivatives of the *Salmonella enterica* serovar Typhimurium (*S*. Tm) streptomycin-resistant wild-type strain SL1344 (Table 2). Bacterial strains and plasmids used for complementation experiment are listed in Table 2. Gene mutations were generated by using the lambda/red homologous recombination [34]. Primers used for construction of *S*. Tm mutants are listed in S1 Table.

**Table 2:** Strains and plasmids used in this study.

**Strain**	**Genotype**	**Reference**
SL1344	Wild-type *S*. Typhimurium, *hisG*	[35]
T141	SL1344 Δ*pmrA*::*cat*	This study
T230	SL1344 Δ*pagP ΔugtL*::*aphT*	This study
T145	SL1344 Δ*ssaV*::*cat*	This study
T216	SL1344 Δ*ssaV* Δ*pagP* Δ*ugtL*::*aphT*	This study
T118	SL1344 Δ*spiB*::*aphT*	[25]
T223	SL1344 Δ*spiB ΔpmrA*::*cat*	This study
T227	SL1344 Δ*ssaV ΔpagP*::*aphT*	This study
T228	SL1344 Δ*ssaV ΔugtL*::*aphT*	This study
T343	SL1344 Δ*ssaV* Δ*pagP* Δ*ugtL*	This study
T364	T343 harboring pMW118 and pACYC184	This study
T365	T343 harboring pT112 and pT115	This study
**Plasmid**	**Genotype**	**Reference**
pMW118	Low-copy number expression vector	Nippon Gene
pACYC184	Middle-copy number expression vector	New England BioLabs
pT112	pMW118 containing a 500 bp from the upstream region and coding region of *pagP*	This study
pT115	pACYC184 containing a 500 bp from the upstream region and coding region of *ugtL*	This study

### Plasmid construction

The complementary plasmid pT112 was constructed using PCR fragments containing a 500 bp from the upstream region and coding region of *pagP* generated with primers PropagP-EcoRV-FW and pagP-SalI-RV and *S*. Tm SL1344 chromosomal DNA as template, which were digested with EcoRV and SalI, and then ligated between the SmaI and SalI sites of pMW118. The complementary plasmid pT115 was constructed using PCR fragments containing a 500 bp from the upstream region and coding region of *ugtL* generated with primers ProugtL-EcoRV-FW and ugtL-SalI-RV and *S*. Tm SL1344 chromosomal DNA as template, which were digested with EcoRV and SalI, and then ligated between the EcoRV and SalI sites of pACYC184. Primers used for construction of the complementation plasmid are listed in S1 Table.

### Determination of minimum inhibitory concentrations (MICs)

The overnight cultures of *S*. Tm strains were diluted and then inoculated onto the 96-well microtiter plates at the starting absorbance value determined at 600 nm (*A*_*600*_) of 0.025, in a total volume of 100 μL Cation-adjusted Mueller-Hinton broth (CAMHB) supplemented with different concentrations of polymyxin B (Wako) and protamine (MP Biomedicals). After 20 h of incubation at 37°C, the *A*_*600*_ values were determined using a microplate reader (Bio-Rad). Each experiment was repeated in duplicate. A positive control contained no CAMPs whereas in the negative control, *S*. Tm cells were not present. MICs were determined as the lowest concentrations of CAMPs that were shown to inhibit bacterial growth by more than 50% in comparison with the growth of the positive control.

### Antimicrobial killing assay (*in vitro* killing assay)

Killing assay was performed as described previously, with minor modification [36]. Briefly, *S*. Tm cells at the mid-logarithmic or the stationary growth phase were washed and resuspended in PBS at the density of 1–5 × 10^7^ CFU/mL. The diluted bacterial suspension was treated with polymyxin B (1 μg/ml; Wako), or protamine (0.5 mg/ml; MP Biomedicals), or magainin 2 (128 μg/mL; LKT Laboratories, Inc.) at 37°C for 30 min. Following the incubation, the mixture was plated on the selective LB medium. The recovered bacterial levels were normalized to those of the control, where PBS was used instead of the antimicrobial peptides, yielding bacterial relative survival values.

### Mice and infection experiments

All mice used in this study are C57BL/6 mice, housed under the specific pathogen-free conditions in the Institute of Animal Experiments of the School of Pharmacy, Kitasato University, Japan. These infection experiments were performed as described previously [37]. Briefly, 6- to 13-week-old mice were deprived of food and water for 4 h, and then inoculated with 25 mg streptomycin sulfate (Wako Pure Chemical Industries) by gavage to attenuate the colonization resistance. Twenty-four hours later, mice were deprived of food and water, and then inoculated with bacteria (1 × 10^8^ CFU) by gavage. To determine the bacterial loads in the colonic content, fresh fecal pellets were freshly collected, and homogenized in sterile phosphate-buffered saline (PBS) for the plating on MacConkey agar plates (Nissui Pharmaceutical) supplemented with the appropriate antibiotic(s). To monitor bacterial loads in luminal content and organs, cecal content, mLN and spleen were recovered from mice sacrificed by cervical dislocation or carbon dioxide inhalation at the indicated time points, and then homogenized in sterile PBS containing 0.5% tergitol and plated on MacConkey agar plates supplemented with the appropriate antibiotic(s).

### Histopathology

Parts of cecal tissue were fixed in 4% formaldehyde (Mildform, Wako Pure Chemical Industries) and embedded in paraffin. Cryosections were prepared, air-dried and stained with hematoxylin/eosin.

### Reverse transcription quantitative (q)PCR

The cecum was excised to a quarter part, and stored in the RLT buffer (Qiagen) at -80°C until further analyses. Total RNA was extracted from homogenized cecal tissue using the RNeasy Mini Kit (Qiagen). Five hundred microgram of total RNA was used for reverse transcription using TaqMan Reverse Transcription Reagents (ThermoFisher Scientific). qPCR was conducted using a CFX96 Real-Time PCR Detection System (Bio-Rad), using the SsoAdvanced Universal SYBR Green Supermix (Bio-Rad) or KAPA SYBR FAST qPCR Master Mix (Kapa Biosystems), according to the manufacturers’ instructions. Relative mRNA expression was calculated using the ΔCt method with *GAPDH* as a reference gene [38]. Utilized primers in the analysis are shown in S1 Table.

### EtBr influx assay

EtBr influx assay was performed as previously described [27]. Briefly, *S*. Tm cells grown to the mid-logarithmic growth phase were washed and resuspended in binding buffer (25 mM MES pH 6.0, 25 mM NaCl), and diluted to the optical density (OD)_600_ of 0.4/mL in the binding buffer. When necessary, the bacterial suspensions were preincubated with CCCP (48 μM) for 10 min. Following the addition of EtBr (24 μM), the fluorescence signal intensity of the EtBr-nucleic acid complex was measured using SpectraMax M5 spectrofluorometer (Molecular Devices) with excitation and emission wavelengths of 544 and 590 nm, respectively.

### Statistical analysis

The exact Mann-Whitney U test was performed using the software GraphPad Prism. P values of less than 0.05 were considered to indicate statistically significance.

### Ethical statement

This study and all animal experiments were reviewed and approved by the Kitasato University Institutional Animal Care and Use Committee (Permit Number: A13-6, J96-1 and J13-1).

## Supporting information

S1 Fig PagP- and UgtL-, or PmrA-dependent resistance to antimicrobial peptides. Wild-type *S*. Tm strain (SL1344), Δ*pagP ugtL*::*kan* strain (T230) or Δ*pmrA*::*cat* strain (T141) from the stationary growth phase was incubated with (A) polymyxin B (1 μg/mL) or (B) protamine (0.5 mg/mL) for 30 min at 37°C. Bacterial survival was quantified by dilution-plating. Bacterial killing effect was evaluated by comparing the sample without the antimicrobial peptides, meaning that less than 1 indicates killing effect was increased. n = 9 (polymyxin B) or 7 (protamine). Horizontal bars, median; ns, not significant (P ≥ 0.05); **P < 0.01; Mann-Whitney U test.
(EPS)

S2 Fig Mutation of *pagP* or *ugtL* gene alone confers no colonization defect. Streptomycin-pretreated C57BL/6 mice (n = 11 or 12) were infected for 7 days with a 1:1 mixture (total 1 × 10^8^ CFU intragastrically) of *ssaV* mutant and *ssaV pagP* mutant, or *ssaV* mutant and *ssaV ugtL* mutant. *S*. Tm loads in feces (A and D), cecal content (B and E), and mLN (C and F) were determined by selective plating. Competitive infection indices (CI) were determined at 1, 3, 5 and 7 days post infection. Horizontal bars, median; ns, not significant (P ≥ 0.05); *P < 0.05; **P < 0.01; ***P < 0.001; Mann-Whitney U test.
(EPS)

S3 Fig Complementation of the *pagP* and *ugtL* mutations restores the attenuated resistance to magainin 2. Δ*ssaV*::*cat* (T145) or Δ*ssaV* Δ*pagP* Δ*ugtL* (T343) or T343 harboring pMW118 and pACYC184 (T364) or T343 harboring pT112 encoding *pagP* (pMW-*pagP*) and pT115 encoding *ugtL* (pACYC-*ugtL*) (T365) from the mid-logarithmic growth phase was incubated with magainin 2 (128 μg/mL) for 30 min at 37°C. Bacterial survival was quantified by dilution-plating. Bacterial killing effect was evaluated by comparing the sample without magainin 2, meaning that less than 1 indicates killing effect was increased. n = 6. Horizontal bars, median; ns, not significant (P ≥ 0.05); **P < 0.01; Mann-Whitney U test.
(EPS)

S1 Table A list of primers used in this study. (XLSX)
